# Clinical and environmental implications of a high-density clip closure after endoscopic submucosal dissection: is it worth it?

**DOI:** 10.1055/a-2717-1584

**Published:** 2025-10-29

**Authors:** Elena De Cristofaro, Jean Grimaldi, Jérémie Jacques, Jérôme Rivory, Edoardo Troncone, Timothée Wallenhorst, Mathieu Pioche

**Affiliations:** 1 60259 Gastroenterology Unit, Department of Systems Medicine, University of Rome Tor Vergata, Rome, Italy; 2 Gastroenterology and Endoscopy Unit, Edouard Herriot Hospital, Hospices Civils de Lyon, Lyon, France; 3 Gastroenterology and Endoscopy Unit, Dupuytren University Hospital, Limoges, France; 4 36684 Gastroenterology and Endoscopy Unit, Pontchaillou University Hospital, Rennes, France

Prophylactic clip closure for the prevention of delayed bleeding after endoscopic submucosal dissection (ESD) remains debated. While some retrospective studies have shown a benefit, recent randomized trials have not confirmed it 1 2 3 4 5 .

These studies often report the use of many clips to achieve closure, emphasizing tight, high-density placement. However, this practice is not standardized, and such variability raises additional concerns regarding procedural costs and environmental impact. In our clinical practice, closure is considered complete when no visible mucosal defect remains after clip placement ( Fig. 1 ).

**Fig. 1:**
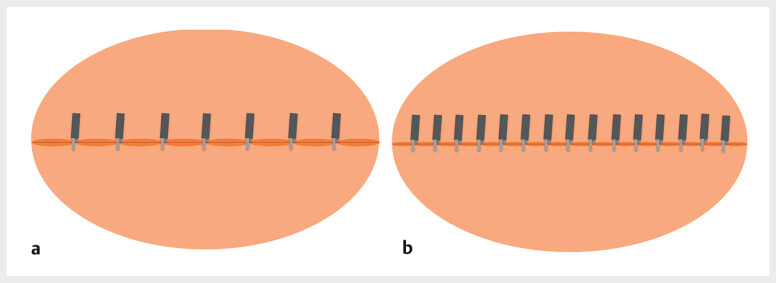
Two different techniques for closing a mucosal defect after endoscopic submucosal dissection. Panel **a** : Complete closure with standard clip placement and without any visible residual mucosal defect. Panel **b** : Complete closure with high-density clip placement.

Here, we present the case of a 76-year-old man referred for endoscopic resection of a large (7 cm), non-granular laterally spreading tumor at the hepatic flexure.

Due to the lesion’s characteristics, ESD was indicated and successfully performed using an adaptive traction device (A-TRACT) in 70 minutes. The Limoges bleeding risk score was 5 points, indicating high risk. Therefore, complete prophylactic clip closure was performed.

Closure was performed using the clip-anchoring technique, with incisions enhancing a clip grip. Departing from our standard practice, we applied a “Japanese-style” closure with <1 mm spacing between clips. This required 30 additional minutes to close a defect of approximately 8 cm, using 15 reloadable clips with a single-use handle ( Video 1 ). The environmental impact was 50.2 g for the first clip with handle, plus 4 g per clip, for a total of 106.2 g and 1.35 kg CO _2_ equivalent. In terms of cost, the use of reloadable clips amounted to a total of 216 euros.

Economic and environmental burden of complete clip closure after colonic ESD.Video 1

In comparison, 15 single-use clips would have produced 1062 g of waste, 10.9 kg of CO _2_ equivalent, and a cost of € 600.

Considering the extra time, costs, and environmental burden, we hypothesize that this technique should only be widely adopted if future randomized trials confirm its clinical benefit over partial or no closure and over other systems and strategies. Until then, “Japanese-style” prophylactic closure should be carefully reserved for selected scenarios.

Endoscopy_UCTN_Code_TTT_1AV

## Notes

Endoscopy E-Videos https://eref.thieme.de/e-videos
*E-Videos* is an open access online section of the journal *Endoscopy* , reporting on interesting cases and new techniques in gastroenterological endoscopy. All papers include a high-quality video and are published with a Creative Commons CC-BY license. Endoscopy E-Videos qualify for HINARI discounts and waivers and eligibility is automatically checked during the submission process. We grant 100% waivers to articles whose corresponding authors are based in Group A countries and 50% waivers to those who are based in Group B countries as classified by Research4Life (see: https://www.research4life.org/access/eligibility/ ). This section has its own submission website at https://mc.manuscriptcentral.com/e-videos .
